# Prevalence of Antimicrobial and Colistin Resistance in Enterobacterales in Healthy Pigs in Ghana Before and After Farmer Education

**DOI:** 10.3390/tropicalmed10090266

**Published:** 2025-09-17

**Authors:** Elvis Fiam Amegayibor, Rita Ohene Larbi, Matilda Ayim-Akonor, Ebenezer D. O. Ansa, Pruthu Thekkur, Helena Owusu, Robert Fraser Terry, Anthony D. Harries, Benjamin Kissi Sasu, George Kwesi Hedidor, Richael Odarkor Mills

**Affiliations:** 1 Department of Biomedical Sciences, School of Allied Health Sciences, University of Cape Coast, Cape Coast PMB UCC Post Office, Cape Coast CC-123-1749, Ghana; 2 Council for Scientific and Industrial Research—Animal Research Institute, Accra P.O. Box AH20, Ghana; 3 International Union Against Tuberculosis and Lung Disease (The Union), 2 Rue Lantier, 75001 Paris, France; 4 The Union South-East Asia Office 9 (The USEA), New Delhi 110016, India; 5 Pharmacy Department, Korle Bu Teaching Hospital, Accra P.O. Box KB77, Ghana; 6 Special Programme for Research and Training in Tropical Diseases, World Health Organization, Avenue Appia 20, 1203 Geneva, Switzerland; 7 Department of Clinical Research, Faculty of Infectious and Tropical Diseases, London School of Hygiene and Tropical Medicine, Keppel Street, London WC1E 7HT, UK; 8 Veterinary Services Directorate, National Food Safety Laboratory (AMR Reference Laboratory for Animal Health), Accra P.O. Box M 161, Ghana; 9 World Health Organization, Country Office Roman Ridge, Accra P.O. Box MB 142, Ghana

**Keywords:** West Africa, farms in Ghana, pigs, antimicrobial use, *Escherichia coli*, *Enterobacter* spp., antimicrobial resistance (AMR), *mcr-1* gene, operational research, SORT IT

## Abstract

High levels of antimicrobial resistance (AMR) were found in healthy pigs in the Greater Accra region of Ghana in 2022; this led to awareness creation and education of pig farmers about how to improve antimicrobial practices and biosecurity. To assess changes in AMR after the intervention, we compared AMR, multi-drug resistance (MDR), and determined colistin resistance levels in healthy pigs in the region before (2022) and after (2024) the education of farmers. Rectal swabs (*n* = 140) from pigs in 14 farms were cultured for isolation of Enterobacterales (*Escherichia coli* and *Enterobacter* spp.) and subjected to antimicrobial susceptibility testing. A selective culture method was employed to isolate colistin-resistant Enterobacterales, which were further screened for the *mcr-1* gene. AMR and MDR findings were compared with those from 140 pigs from the same farms in 2022. Enterobacterales were found in 137 (2022) and 138 (2024) rectal swabs, with *Escherichia coli* predominating. There was a marked increase in AMR prevalence for eight of the nine tested antibiotics in 2024. Notable increases in resistance were for tetracycline (58–82%), ampicillin (33–49%), and ciprofloxacin (3–33%). MDR prevalence was highest in *Escherichia coli*, increasing from 20% to 44%. Phenotypic colistin resistance was found in 44% of *Escherichia coli* isolates in 2024 (in 2022, using different methodology, phenotypic colistin resistance was 8%)—in 2024, the *mcr-1* gene was found in 51% of these isolates. There were no relevant associations between farm and pig characteristics and prevalence of MDR and phenotypic colistin resistance. Although education is an important aspect of AMR control, the findings highlight that education alone cannot curb the rise of AMR. Further interventions including continuous surveillance, stronger regulatory policy on antimicrobial use, and behavioral interventions will be needed to mitigate the situation.

## 1. Introduction

Gram-negative Enterobacterales, that are antimicrobial-resistant (AMR), multidrug resistant (MDR), and colistin resistant, have been increasing across Africa, particularly in West Africa including Ghana [1,2,3]. MDR Enterobacterales of animal origin, including diarrheagenic *Escherichia coli* (*E. coli*), have been implicated in human food-related outbreaks whereas difficulty in treating extended spectrum beta-lactamase and colistin resistant Enterobacterales (*E. coli*, *Klebsiella*, *Salmonella* and *Enterobacter* spp.) has resulted in prolonged hospital stays and mortality [4,5].

This threat to public health is largely attributable to extensive use and abuse of antimicrobial agents in both humans and animal husbandry. This includes pigs, that are frequently consumed in restaurants and on special occasions [6].

MDR (defined as resistance to three or more antimicrobial classes) [7] is especially prevalent in Enterobacterales. In the last few years, there has been a significant increase in MDR-Enterobacterales in animals, and this constitutes both a direct and indirect risk to food safety with regard to human and public health [8]. Colistin (or polymyxin E) is an antimicrobial that is recognized by the World Health Organization (WHO) as a last resort treatment option for MDR gram-negative bacterial infections in humans. Since its introduction on the market in the 1960s, colistin has been used in pig production farms in several countries for the treatment and prevention of infection and the promotion of growth [9]. These practices can in time lead to the development of resistance, which may occur either through chromosomal mutation or because of transfer of plasmid borne genes between bacteria. Plasmid borne genes (*mcr-1* to *mcr-10*) encode for colistin-resistance [10], and these constitute a further public health threat as they enable horizontal transfer of resistance from gram-negative bacteria to other bacteria, further disarming the efficacy of this last resort drug.

There have been a few studies in Africa reporting high levels of MDR Enterobacterales in pigs and pork [2,3,11]. A dearth of animal-based studies, and therefore large knowledge gaps, hinder the development of effective evidence-based One Health policies on AMR. Similarly, while colistin has been used in animal farming in Africa for years, prompting concerns that the pig rearing industry in this region may be a reservoir for bacteria resistant to colistin, there are few studies about colistin resistance in animals in African countries [12].

Against this background, Ohene Larbi and colleagues conducted a study in 2022 on healthy pigs in commercial farms in the Greater Accra region of Ghana; this was focused on Enterobacterales and their resistance profiles to antimicrobials which are frequently used in veterinary and human medicine [13]. The two main Enterobacterales identified in the pig rectal swabs were *Escherichia coli* (*E. coli*) and *Enterobacter* spp., with the prevalence of MDR being found at 23% and 5%, respectively. Phenotypic resistance to colistin was also found in 8% of both *E. coli* and *Enterobacter* spp. isolates, with molecular resistance (detected by the presence of the *mcr-1* gene) found in half of the isolates. All the farms used antimicrobials for treatment/prophylaxis, these being predominately tetracyclines, penicillins, and aminoglycosides, although farmers stated they did not use antimicrobials in farm feed to promote growth.

Following the study, the authors developed a stakeholder list that prioritized important policy and decision makers to whom the dissemination and the key findings were directed. Dissemination tools included a plain language policy brief, short (3 min) and long (10 min) power point presentations and an elevator pitch, all of these developed during a Structured Operational Research Training Initiative (SORT-IT) course workshop held in October 2022 [14]. Several recommendations were made that included the following: (i) education of the farmers and livestock handlers about human and animal hygiene practices, biosafety and biosecurity measures, proper antimicrobial use, and how to ensure good animal husbandry; (ii) further and more detailed assessment of how antimicrobials are used on the farms; (iii) strengthening of regulatory policy to curb the practice of using antimicrobials as growth promoters in the animal industry; and (iv) further research into the presence of antimicrobial residues in farm feeds and environmental samples such as dust and water. The dissemination activities and the recommendations made are shown in detail in Supplementary Materials S1.

Following these recommendations, particularly about education of farmers on proper antimicrobial use, we anticipated that there might be changes in the AMR, MDR, and colistin-resistant patterns in healthy pigs alongside changes in antimicrobial usage and hygiene practices on the farms. A follow-up study was therefore proposed and implemented. The study aimed to compare AMR, MDR, and colistin-resistance patterns in *E. coli* and *Enterobacter* spp. in healthy pigs in selected farms in the Greater Accra region of Ghana before (January–March 2022) and after (August–November 2024) farmer education about antimicrobial practices. The specific objectives were as follows: (i) compare in 14 selected farms in the Greater Accra region of Ghana between 2022 and 2024, characteristics of healthy pigs and their farms, the prevalence of pig rectal swabs with *E. coli* and *Enterobacter* spp. and the prevalence of *E. coli* and *Enterobacter* spp. isolates with AMR, MDR, and phenotypic and molecular colistin-resistance; and (ii) determine characteristics of pigs and farms associated with MDR and phenotypic colistin-resistance in 2024.

## 2. Materials and Methods

### 2.1. Study Design

This was a comparative cross-sectional study using primary data.

### 2.2. General Setting

Ghana is a country located along the Gulf of Guinea and the Atlantic Ocean, in the subregion of West Africa. Ghana has a population of approximately 30.8 million, and Accra is the capital city with a population of 5.4 million [15].

### 2.3. Site-Specific Setting

There are 29 districts within the Greater Accra region of Ghana [15]. Information acquired from the Greater Accra Pig Farmers Association and the Ministry of Food and Agriculture (MOFA) indicated that there were four districts (Adenta, Ningo-Prampram, Shai-Osudoku, and Ga South) with large numbers of commercial pig farms. These districts were selected for the first study in 2022. In that study, five pig farms within each district were randomly selected, making a total of 20 farms. This new study in 2024 was conducted in the same four districts. However, due to economic constraints, six of the farms discontinued pig production at the time of the study, hence 14 of the farms used in the previous study [13] were reassessed for the current study.

### 2.4. Sampling

Visits to the farms and collection of samples followed the same methodology as described by Ohene Larbi et al. in the previous study [13]. Visits were done on a district-by-district basis to the 14 farms that were assessed previously with the study team consisting of a veterinarian, a field assistant, a Livestock Extension Officer of MOFA, and the principal investigator. At each farm, the team briefed the farmer on the purpose and details of the study. Once the farmer agreed to participate, a consent form was given to the farmer to either sign or thumb print. A questionnaire developed by using an open-source tool (Epicollect5) application was administered to obtain information about the farm and pigs and use of antimicrobials, and GPS coordinates for each farm were also collected. This was the same questionnaire as used in the 2022 study, which had been pilot-tested and validated before use. In most cases, the questions were explained in a local language (Twi or Ga) to allow for clarity in answers to the questions.

With the help of the veterinarian, 10 apparently healthy pigs from each farm, weighing between 20 kg and 50 kg, that had passed the weaning stage, and were below 12 months in age, were selected based on sampling methodology related to the number of pens (from prior experience, the number of pens varied from 3 to 10 per farm) There was a random selection of pigs from each farm, and on each selected farm, pigs were selected from two or more pens. Apparently healthy pigs were studied rather than unhealthy pigs because they constitute the main source of food for human consumption [16]. Unhealthy pigs were identified and excluded from the study as a result of inspection by the veterinarian.

The veterinarian took one rectal swab from each pig using a sterile cotton swab stick; the swabs were collected from August to November 2024. Rectal swabs were placed in 1 mL buffered peptone water and then transported on ice to the microbiology laboratory of the Centre for Scientific and Industrial Research (CSIR)—Animal Research Institute for further processing within 24 h of sample collection.

### 2.5. Study Population

The study population included ten apparently healthy pigs from each of the 14 farms in 2024: in total, there were 140 pigs. The microbiological findings were compared with 140 apparently healthy pigs examined from the same 14 farms in the first study in 2022.

### 2.6. Specimen Processing and Laboratory Procedures

#### 2.6.1. Bacterial Isolation

On arrival at the laboratory, each rectal swab in its 1 mL buffered peptone water was incubated for 18–24 h at 37 °C (incubation conditions remained constant for all procedures). A loopful of each incubated sample was then plated out on MacConkey agar (Oxoid Ltd., Basingstoke, Hampshire, UK) and incubated. Sub-cultures were made on Eosin Methylene Blue agar for bacterial colonies that exhibited distinct colonial morphology of *E. coli* and *Enterobacter* spp., (green metallic sheen or blue black colonies) [17], and these were subsequently incubated under similar conditions. For further confirmation, biochemical tests for sulfur, indole, motility, citrate, and Triple Sugar Iron, were conducted [18].

#### 2.6.2. Antimicrobial Susceptibility Testing

Using the Kirby Bauer disc diffusion method, antimicrobial susceptibility tests were conducted in line with the Clinical Laboratory Standards Institute (CLSI) guidelines [19]. The following antimicrobials from their respective classes as grouped by the CLSI (Oxoid Ltd., Basingstoke, Hampshire, UK) were employed: ampicillin 10 μg (penicillins), aztreonam 30 μg (monobactams), amoxicillin/clavulanic acid 30 μg (beta-lactam combination agents), tetracycline 30 μg (tetracyclines), trimethoprim–sulfamethoxazole 25 μg (folate pathway antagonists), chloramphenicol 30 μg (phenicols), ceftazidime 30 μg (cephems), ciprofloxacin 5 μg (fluoroquinolones), and gentamicin 10 μg (aminoglycosides). Each of the nine antibiotics belonged to a different class. Six of the antimicrobials (ampicillin, tetracycline, amoxicillin/clavulanic acid, trimethoprim–sulfamethoxazole, ciprofloxacin, and gentamicin) fall within the veterinary critically important antimicrobial agents (VCIA) category of the World Organisation for Animal Health [20]. The three other antibiotics (chloramphenicol, aztreonam, and ceftazidime) are recognized as highly important antimicrobials for use in humans [21]. For quality control, the reference strain *E. coli* ATCC 25922 was used. Definition of MDR by Magiorakos et al. as any isolate showing resistance to three or more antimicrobial classes was applied to identify MDR isolates [7]. When intrinsic resistance to a specific antimicrobial was present in bacteria, susceptibility tests were not performed.

There were two methods used to determine whether the bacteria were resistant to colistin. The first method used in the 2022 study was to screen *E. coli* isolates from MacConkey agar whereby overnight cultures on Nutrient agar (Neogen Corporation; 620 Lesher Place, Lansing, MI, USA) were set to a 0.5 McFarland suspension and a loopful of each then plated on CHROMagar COL-APSE [22]. The second method, which was introduced in 2024 as an improvement of the 2022 study, was to plate all processed pig rectal samples directly on CHROMagar COL-APSE. Pink and blue isolates representing presumptive *E. coli* and *Enterobacter* spp. were subjected to similar biochemical tests as mentioned above.

#### 2.6.3. Molecular Detection of *mcr-1* Gene

All colistin resistant isolates from both methods were analyzed molecularly to determine the *mcr-1* gene using the primer CLR5-F (5′CGGTCAGTCCGTTTGTTC-3′) and CLR5-R (5′-CTTGGTCGGTCTGTA GGG-3′). Polymerase chain reactions (PCR) were performed after extracting bacterial DNA by the boiling method [2,3]. DNA quality and concentration were determined using a SimpliNano spectrophotometer. Nuclease free water and an in-house *mcr-1* positive strain were used as negative and positive controls. See Figure S1 in Supplementary Materials S2. The total reaction volume was set to 25 μL by the addition of 5 μL of 2× master mix, 1 μL each of forward and reverse primer, 6 μL of DNA template, and 12 μL of double distilled water. The optimal conditions for PCR amplification of the *mcr-1* gene were 94 °C for 2 min; 40 cycles of denaturation at 94 °C for 1 min; annealing at 59 °C for 1 min; extension at 72 °C for 2 min; final extension at 72 °C for 10 min; and holding at 4 °C for infinity.

As part of quality assurance, the laboratory data were sent to the National AMR Reference Laboratory-Animal Health as part of the annual entry into the International FAO Antimicrobial Resistance Monitoring System (InFarm).

### 2.7. Data Variables and Sources of Data

Variables included the following: Farm and pigs—pig age and sex; farm annual production turnover; type of farm feed and any change in source of feed from previously; addition of antimicrobials to feed (for instance tetracycline or colistin); antimicrobials given for treatment in the last 12 months; antimicrobials given for prophylaxis in the last 12 months; frequency of antimicrobials used in prophylaxis in the last 12 months. Bacterial isolates—*E. coli* and *Enterobacter* spp. obtained from rectal swabs; for each *E. coli* and *Enterobacter* spp. isolate, antimicrobial resistance (defined as intermediate or resistant) or susceptibility to ampicillin, aztreonam, amoxicillin/clavulanic acid, tetracycline, trimethoprim–sulfamethoxazole, chloramphenicol, ceftazidime, ciprofloxacin, and gentamicin. (A diagnosis of MDR was made if an isolate showed resistance to three or more of the antimicrobial classes; in this definition, colistin was omitted.) Colistin—phenotypic-sensitive or phenotypic-resistant; genotypic resistance based on the detection of the *mcr-1* gene.

### 2.8. Data Analysis

Pig and farm data were collected using Epicollect5, a free application made for questionnaire development and field data collection with or without internet connection [23]. Identity numbers generated for the ten pigs on each farm were linked to their farm characteristics and to the laboratory data generated at the CSIR Animal Research Institute Laboratory. This data was extracted into MS Excel and exported to Jamovi version 2.3.28 (2024) for further cleaning and analysis.

A descriptive analysis was undertaken to document frequencies and proportions from categorical variables. Characteristics of the pigs, their farms, and rectal swabs with isolates of *E. coli* and *Enterobacter* spp., AMR and MDR patterns, and phenotypic colistin resistance were compared between pigs in 2022 and pigs in 2024 using the chi-square test and Mann–Whitney test for categorical and continuous variables, respectively. Pig and farm characteristics associated with MDR and phenotypic colistin resistance in 2024 were assessed using log-binomial regression after adjusting for clustering at the farms. Prevalence ratios (PR) and 95% confidence intervals were presented as measures of association. Levels of significance were set at 5% (*p* < 0.05).

## 3. Results

### 3.1. Characteristics of the Pigs, Farms, and Antimicrobials Used in the Greater Accra Region of Ghana in 2022 and 2024

The characteristics of the 14 farms and 140 pigs from 2022 and 2024 are shown in Table 1. The proportion of males to females remained fairly similar between the two years. The median age of the pigs remained the same at 4 months. In terms of annual production turnover, large production farms (>300) reduced from 3 to 1 (21.4% to 7.1%), whereas one farm moved from small (≥100) to medium (101–300) production. Also, the number of farms using self-made feed declined from 12 (85.7%) in 2022 to 10 (71.4%) in 2024, with an increase in those using both commercial and self-made feed. Antimicrobial use remained high but reduced by one farm which had not administered any antimicrobials to its animals.

**Figure 1:**
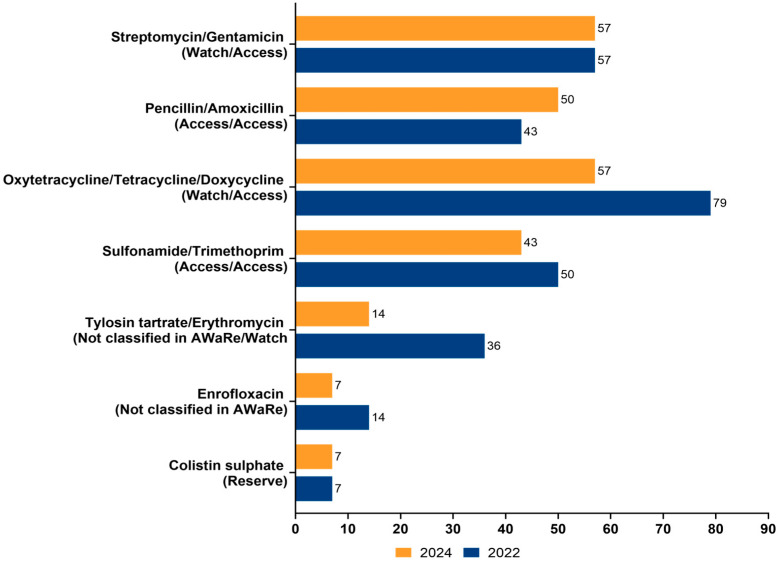
Antimicrobials (grouped in their different classes) used on the 14 pig farms in the Greater Accra Region of Ghana in 2022 and 2024, along with their respective WHO AWaRe classification.

The antimicrobials used on the 14 farms in 2022 and 2024, categorized by their active ingredients and their corresponding WHO AWaRe (Access, Watch, and Reserve) classifications are shown in Figure 1. The AWaRe classification provides insights into proper antibiotic stewardship: Access includes antibiotics that should be widely available and used for common infections; Watch includes antibiotics that require careful monitoring due to their higher potential for resistance; Reserve incudes antibiotics that should be preserved for use in cases where other options are ineffective. For two classes of antimicrobials (colistin and streptomycin/gentamicin), there was no change. For four classes there was a decrease in use, which was especially marked for tetracyclines which decreased from 78.6% to 57.1%. In contrast, the administration of penicillin and amoxicillin experienced a slight increase in use from 42.9% to 50%.

### 3.2. Enterobacterales of Healthy Pigs in 2022 and 2024

The isolation rate of Enterobacterales in pig samples remained consistently high at above 97% in both 2022 and 2024. The proportion of samples containing only *E. coli* significantly increased from 85.4% to 94.2%. Similarly, the proportion of samples harboring both *E. coli* and *Enterobacter* spp. significantly decreased from 14.6% to 5.8%. These results are shown in Table 2.

### 3.3. AMR and Colistin Resistance of Enterobacterales

**Figure 2:**
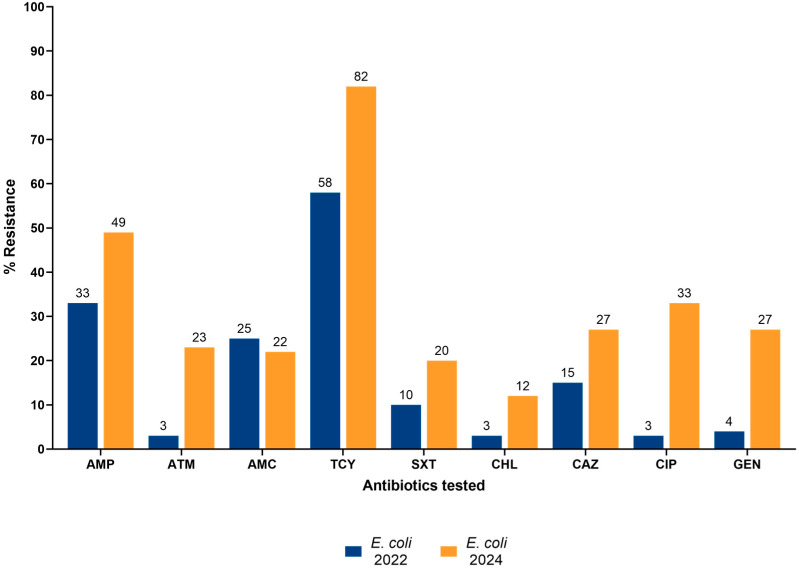
Antimicrobial resistance patterns of *E. coli* in healthy pigs in the Greater Accra Region of Ghana: 2022 and 2024. Footnotes: AMP: Ampicillin; ATM: Aztreonam, AMC: Amoxicillin clavulanic acid; TCY: Tetracycline; SXT: Trimethoprim–sulfamethoxazole; CHL: Chloramphenicol; CAZ: Ceftazidime; CIP: Ciprofloxacin; GEN: Gentamicin.

The levels of AMR in *E. coli* isolates from 2022 and 2024 are shown in Figure 2. Overall, there was a marked increase in the prevalence of AMR for all tested antibiotics in 2024 except amoxicillin/clavulanic acid, which showed a slight decrease from 24.8% in 2022 to 21.7% in 2024. The highest increase was observed in ciprofloxacin resistance, which rose from 2.9% in 2022 to 33.3% in 2024. Resistance to tetracycline and ampicillin which were highest in 2022 remained high in 2024 at 81.9% and 49.3%, respectively. Resistance to gentamicin, chloramphenicol, and trimethoprim–sulfamethoxazole, which were lower than 5% in 2022, all increased to above 10% in 2024.

The distribution of MDR *E. coli* isolates obtained from rectal swabs of healthy pigs in the Greater Accra Region of Ghana in 2022 and 2024 is summarized in Table 3. The number of *E. coli* isolates showing MDR significantly increased from 19.7% in 2022 to 44.2% in 2024. In 2022, the patterns of MDR resistance were seen for three, four, and five classes of antimicrobials. In 2024, there was a much broader range of MDR resistance, with 34 (56%) of the MDR isolates showing resistance to three, four, or five classes of antimicrobials and 27 (44%) showing resistance to between six and nine classes of antimicrobials. For *Enterobacter* spp., 1 of 21 isolates in 2022 showed MDR while none of 8 isolates in 2024 showed MDR.

In 2024, out of 140 samples screened directly for phenotypic resistance to colistin, 43.6% of *E. coli* and 3.6% *Enterobacter* spp. showed resistance to colistin. Almost half, 49.2% of *E. coli,* had the *mcr-1* gene. The results are shown in Table 4. Comparisons with 2022 are not presented because of the different laboratory methodologies used.

### 3.4. Characteristics Associated with MDR and Colistin Resistance in 2024

Factors associated with MDR in 2024 are shown in Table 5. In 2024, out of 138 pig rectal samples with *Enterobacteriaceae*, 61 (44.2%) were identified as having MDR, with a higher MDR prevalence in female pigs (51.4%). Farm practices influenced MDR, with significantly higher MDR prevalence being found on farms producing 30–100 pigs annually (66.0%) or >300 pigs annually (60%) and on farms using a combination of commercial and self-made feed (80.0%). Change of farm feed in 2024 was also associated with an increased prevalence of MDR (*p* = 0.004). There were no associations between antimicrobial use for treatment and prophylaxis or use of veterinary consultation with MDR.

Characteristics associated with phenotypic resistance to colistin are shown in Table 6. Colistin resistance was present in 47.1% of 140 samples screened. The significant findings were a higher prevalence of colistin resistance in pigs aged 8–12 months (71.4%), where there had been a change in farm feed (60.0%), where antimicrobials had been used for prophylaxis (56.0%), and where no antimicrobials had been used (70.0%). There were no other associations.

## 4. Discussion

The key finding in this operational research study in the Greater Accra region of Ghana was the observed increase in AMR and MDR amongst Enterobacterales in healthy pigs in 2024 compared with 2022. As with the first study in 2022 [13], and consistent with other studies that have examined fecal bacteria in healthy pigs [9,24,25], the predominant species in the rectal swabs of pigs was *E. coli*. Eight of the nine tested antibiotics, apart from amoxicillin/clavulanic acid, showed a marked increase in AMR to *E. coli*, with particularly large increases in resistance being found with ampicillin, tetracycline, and ciprofloxacin. The prevalence of MDR in *E. coli* isolates more than doubled in the two years from 19.7% to 44.2%, with almost half of these showing resistance to six or more antibiotics in contrast to no such patterns in 2022. This high prevalence of resistance, however, is at a similar level of magnitude when compared with another study in the Greater Accra region in 2022–2023 where *E. coli* isolates from 200 healthy cattle and 100 healthy pigs showed MDR prevalence of 55% and 35%, respectively, with high levels of resistance also to ampicillin and tetracycline [26]. Finally, just over 40% of *E. coli* isolates showed phenotypic resistance to colistin. We could not perform direct comparisons with 2022 as different methodologies for assessing phenotypic colistin resistance were used. Nevertheless, there appeared to be a large increase during the two years with phenotypic resistance to colistin being 7.7% in 2022 [13].

So, why was there a large increase in AMR, MDR, and colistin resistance in this two-year period? Farmers and livestock handlers received education at a One Health Farmers Meeting and at Face-to-Face meetings about human and animal hygiene practices, biosafety and biosecurity measures, proper antimicrobial use, and good animal husbandry. This should have improved farmers’ knowledge about AMR, but the study did not assess the quality of these education sessions nor the farmers’ knowledge through questionnaires after the education sessions. This was a missed opportunity. A recent study in Ghana has shown that although the majority of farmers use antibiotics, their knowledge about antibiotic use and AMR is poor [27]. Almost all farmers purchase their antibiotics from markets, the majority do not keep records of antibiotic use and only half of farmers understand the concept of withdrawal periods (defined as the length of time for antibiotic concentrations in animal tissues to reduce to safe levels after treatment or prophylaxis is completed) [27].

Despite the education sessions, there were no significant changes in pig and farm characteristics and practices between 2022 and 2024, and antimicrobial use remained high. Using the WHO AWaRe classifications for classes of antimicrobials, colistin use remained the same, tetracycline use declined only slightly, and penicillin/amoxycillin use increased. We realized that after the education sessions, some of the attending farm hands vacated the farms and were replaced by new staff, which would have left a knowledge gap on the prudent use of antimicrobials and proper hygiene and management practices. However, even on farms with no change of staff, management practices that involved better maintenance of functional footbaths, proper fecal waste management and cleanliness of farm equipment were poorly observed and remained the same. Such lack of improvement is usually blamed on costs. However, within the agricultural sector, there is a growing body of evidence suggesting a disconnect between knowledge and behavior change with respect to biosecurity issues. A study in poultry farms in Ghana showed an important knowledge–behavior gap, with financial constraints, challenges in adapting recommended practices to the local context, limited resources, prioritizing short-term over long-term gains, and underestimating disease risks, all of which can prevent consistent implementation of best practices [28].

There were some farm characteristics and practices in 2024 associated with an increased prevalence of MDR including both low and high pig production, changed farm feed, and the use of both commercial and self-made farm feed, but none of these really explains the observed findings. In particular, the data obtained on antimicrobial use for treatment, prophylaxis, and frequency of prophylaxis showed no association with MDR. Similarly, for phenotypic colistin resistance, the factors associated with an increased prevalence, such as older pigs, changed farm feed, antimicrobial prophylaxis, and use of no antimicrobials, produce a confusing rather than a helpful picture.

Although we did not find an association between lack of veterinarian consultation and MDR, three studies in Ghana in the last 10 years showed that farmers only consult veterinarians when their animals do not respond to treatment, with less than a quarter asking for advice before administering antibiotics themselves [27,29,30]. Where farmers do consult veterinarians before antibiotic administration, there appears to be an improvement in antimicrobial practices [27]. Currently, there is at least one veterinarian and several para-veterinarians in each administrative district of Ghana, all of whom can provide services to farmers and their livestock. Better use needs to be made of this potential source of advice. Help on managerial and technical skills, including better hygienic practices, can also be obtained from Agricultural Extension Officers, although undisclosed high rates of charges by this officer cadre results in less access to this service than might be expected [31].

Another factor that most likely contributed to the increase in AMR and MDR is the widespread and continued administration of broad-spectrum antimicrobials to pigs on farms. Post-weaning pigs often suffer from respiratory and diarrheal disease, and while these may not always be due to infectious agents, farmers nevertheless tend to treat all cases with antimicrobials [32]. Similarly, farmers will often administer antibiotics when animals feed poorly or exhibit stunted growth with some scientific evidence showing that these practices can improve growth performance, lower diarrhea incidence, and improve the humoral immune response [33]. Pig fecal waste is another potential reservoir for resistant bacteria as the raw manure is kept and used on the farm as fertilizer for plants and crops providing ideal conditions for the survival and proliferation of MDR organisms [34]. Fecal manure, containing bacteria and other chemical contaminants such as antibiotic residues, can then leach into the environment and into water sources, and then be further spread through heavy rainfall and other animals such as free-roaming poultry. Alarmingly, fecal waste facilitates horizontal gene transfer, allowing resistance traits to spread between different bacterial species [34]. Current waste management practices fail to address this issue, allowing resistance genes to persist and potentially spread to surrounding ecosystems.

The sustained prevalence of colistin resistance in *E. coli* isolates in 2024, even without increased colistin use, is largely driven by the stable dissemination of the plasmid-borne *mcr-1* gene through horizontal gene transfer across bacterial populations [35]. Once established, these resistant strains can persist in farm environments, livestock, and waste systems, creating self-sustaining reservoirs of resistance independent of direct colistin selection pressure [36]. This genetic linkage may explain why resistance persists despite no increase in colistin use.

There were some strengths to this study. In both 2022 and 2024, the same farms, the same selection criteria for pigs, the same number of pigs, and apart from phenotypic colistin resistance testing where the new method was believed to be better at determining phenotypic resistance [37], the same microbiology processes were used. The study was conducted and reported in accordance with STROBE guidelines (Strengthening the Reporting of Observational Studies in Epidemiology) [38].

However, there were also some limitations. There was no documentation on the farms about whether the individual pigs included in the study received antibiotics for treatment or for prophylaxis, and this might have been important in the genesis of their AMR and MDR. The method used in 2024 to determine phenotypic colistin resistance by plating all processed pig rectal samples directly on CHROMagar COL-APSE was thought to be better than the method of isolate testing used in 2022, but as sample testing increases resistance prevalence compared with isolate testing [35], this prevented a direct comparison of results. We recognize this as an important limitation of the comparative study. We also only assessed whether the *mcr-1* gene was present in samples that showed phenotypic colistin resistance, and we did not determine whether the *mcr-1* gene was present in samples phenotypically sensitive to colistin. Although this was due to resource constraints, it was a missed opportunity as correlation between phenotypic resistance and the presence of the *mcr-1* gene can be weak due to various factors including weak plasmid promoters influencing the expression of genes [39,40,41]. We also did not analyze the other *mcr* genes which can give rise to molecular resistance to colistin. For these reasons, there may therefore have been a higher prevalence of molecular resistance to colistin than we have documented. Finally, clear causal inference to identify risk factors for MDR could not be drawn due to confounding variables, and more information in the future could come from longitudinal studies.

Moving forward, the findings of our study are of concern. A study in China showed that the banning of colistin as a growth promoter in animals resulted in significant decreases in colistin-resistant *E. coli* in pigs and chickens [42]. To make a real difference and halt the spread of AMR, MDR, and colistin resistance in the country, Ghana must strengthen its regulatory policy (e.g., laws regulating antimicrobial use), reduce the indiscriminate use of antimicrobials without veterinary guidance, and consider banning the use of colistin and other antimicrobials, such as tetracycline, as growth promoters. This must be accompanied by bottom-up approaches focused on biosecurity which in turn should reduce the need for antimicrobials by mitigating disease risk and limiting AMR transmission. Training, refresher training, and awareness-raising initiatives are needed, but at the same time farming practices must improve and be monitored. Measures such as the presence of functioning foot baths, wearing separate clothing for work, proper disposal of pig fecal waste, and good record keeping of antimicrobials purchased and used for treatment and prophylaxis must be put in place and quality controlled through regular supervision. Effective waste management strategies are especially important and should be employed to prevent environmental contamination of neighboring households. Without top-down and bottom-up approaches being simultaneously implemented, there is a definite risk of AMR and MDR in Ghana becoming progressively worse.

To better understand the genetic factors that underpin AMR in Ghana, it would be prudent to investigate the genomic epidemiology of AMR to commonly used antibiotics on animal farms and assess how this evolves overtime. Whole genome sequencing would be helpful here. Work has already started on animal farms to detect the genes responsible for penicillin, sulfonamide, and tetracycline resistance [26], and this needs to be continued and expanded to include other antimicrobials. AMR is one of the important global health challenges for the 21st century, with the number of AMR-associated deaths predicted to increase to 8.2 million by 2050 [43] unless concerted action such as minimizing the inappropriate use of antibiotics in farming and humans, takes place. There is no time to be lost.

## 5. Conclusions

Despite increasing the awareness of farmers and conducting education sessions amongst farmers about antimicrobial practices on pig farms, AMR, MDR, and colistin resistance among Enterobacterales in pigs increased between 2022 and 2024. These findings pose serious food safety and One Health risks and highlight gaps in intervention effectiveness and the need for stricter regulatory policy, more prudent antimicrobial use, reinforcement of AMR surveillance programs and stronger behavioral change amongst farmers.

## Figures and Tables

**Table 1:** Characteristics of pigs, their farms, and antimicrobial use in the Greater Accra Region of Ghana: 2022 and 2024.

Characteristics		2022		2024		*p* Value ^#^
		*n*	(%) *	*n*	(%) *	
Pigs	Total	140		140		
Sex						
	Male	75	(53.6)	71	(50.7)	0.635
	Female	65	(46.4)	69	(49.3)	
Age in months	Median (IQR)	4	(3–5)	4	(3–5)	0.739
						
Farms	Total	14		14		
Annual production turnover						
	1–100	6	(42.9)	5	(35.7)	0.410
	101–300	5	(35.7)	8	(57.2)	
	>300	3	(21.4)	1	(7.1)	
Source of farm feed						
	Commercial feed	0	0	1	(7.1)	0.501
	Self-made feed	12	(85.7)	10	(71.5)	
	Both	2	(14.3)	3	(21.4)	
Antimicrobial use in last 12 months						
	Yes	14	(100)	13	(92.9)	0.500
	No	0	0	1	(7.1)	

* Column percentage; # *p* value calculated using the chi-square test for categorical variables and Mann–Whitney test for comparing median age.

**Table 2:** Distribution of Enterobacterales in pigs in the Greater Accra Region of Ghana in 2022 and 2024.

Variable	2022		2024		*p*-Value
	*n*	(%)	*n*	(%)	
Total pig rectal swabs	140	(100)	140	(100)	
Enterobacterales					
Present	137	(97.9)	138	(98.6)	0.999
Absent	3	(2.1)	2	(1.4)	
Type of Enterobacterales					
*Escherichia coli* only	117	(85.4)	130	(94.2)	0.017
*Enterobacter* spp. only	0	(0)	0	(0)	-
*Escherichia coli* and *Enterobacter* spp.	20	(14.6)	8	(5.8)	0.018

**Table 3:** Multi-drug resistant *E. coli* isolates obtained from pig rectal swabs in the Greater Accra Region of Ghana, 2022 and 2024.

Variable	2022		2024	
	*n*	(%)	*n*	(%)
Total	137	(100)	138	(100)
No MDR	110	(80.3)	77	(55.8) **
MDR	27	(19.7)	61	(44.2) **
Resistance to 3 antimicrobial classes	14	(10.2)	18	(13.0)
Resistance to 4 antimicrobial classes	7	(5.1)	8	(5.8)
Resistance to 5 antimicrobial classes	6	(4.4)	8	(5.8)
Resistance to 6 antimicrobial classes	0	(0)	10	(7.2)
Resistance to 7 antimicrobial classes	0	(0)	12	(8.7)
Resistance to 8 antimicrobial classes	0	(0)	3	(2.2)
Resistance to 9 antimicrobial classes	0	(0)	2	(1.4)

Footnotes: Multi-drug resistance = resistance to ≥3 antimicrobial classes: ** *p* < 0.001.

**Table 4:** Colistin resistant isolates of Enterobacterales of pigs in the Greater Accra Region of Ghana in 2024.

Variable	*n*	(%)
Total samples	140	
Phenotypic Colistin resistance		
*E. coli*	61	(43.6)
*Enterobacter* spp.	5	(3.6)
Molecular Colistin resistance *		
*E. coli*	31	(50.8) **
*Enterobacter* spp.	2	(40.0) **

* Defined as presence of *mcr-1* gene; ** denominator = number of *E. coli* and *Enterobacter* spp. showing phenotypic colistin resistance.

**Table 5:** Characteristics associated with MDR of Enterobacterales in pigs in the Greater Accra Region of Ghana in 2024.

Variable	Total	MDR Present *		PR	95% CI	*p* Value
		*n*	(%)			
Pig Characteristics						
Total	138	61	(44.2)			
Sex						
Male	70	26	(37.1)	1		
Female	68	35	(51.5)	1.4	(1.0–2.0)	0.094
Age in months						
2–4 months	99	44	(44.4)	1.2	(0.7–2.2)	0.446
5–7 months	25	9	(36.0)	1		
8–12 months	14	8	(57.1)	1.7	(0.8–3.3)	0.158
Farm Characteristics						
Annual production turnover						
30–100	50	33	(66.0)	2.3	(1.6–3.5)	<0.001
101–300	78	22	(28.2)	1		
>300	10	6	(60.0)	2.1	(1.2–4.0)	0.017
Source of farm feed						
Commercial	10	6	(60.0)	1.9	(1.1–3.4)	0.032
Self-made	98	31	(31.6)	1		
Both	30	24	(80.0)	2.5	(1.8–3.6)	<0.001
Change of farm feed from 2022						
Yes	50	30	(60.0)	1.7	(1.2–2.5)	0.004
No	88	31	(35.2)	1		
Antimicrobial usage						
For treatment only	59	23	(39.0)	1		
For prophylaxis only	49	21	(42.9)	1.1	(0.7–1.7)	0.683
For both prophylaxis and treatment	20	11	(55.0)	1.4	(0.9–2.4)	0.185
No antimicrobials for prophylaxis or treatment	10	6	(60.0)	1.5	(0.9–2.8)	0.158
Frequency of prophylaxis *						
Monthly	40	23	(57.5)	1.2	(0.7–2.2)	0.385
Every two months	20	9	(45.0)	1		
Vet consulted for treating sick pigs *						
Yes	39	15	(38.5)	1		
No	40	19	(47.5)	1.2	(0.7–2.1)	0.421

Footnotes: MDR = multi-drug resistance; PR = prevalence ration; CI = confidence intervals. Vet = veterinary officer. * not all farms indicated frequency of prophylaxis or whether a veterinary officer treated sick pigs and therefore numbers do not add up to 138.

**Table 6:** Characteristics associated with phenotypic colistin resistance in pigs in the Greater Accra region, 2024.

Variable	Total	Colistin Resistance		PR	95% CI	*p* Value
		*n*	(%)			
Pig Characteristics						
Total	140	66	(47.1)			
Sex						
Male	71	39	(54.9)	1.4	(1.0–2.0)	0.065
Female	69	27	(39.1)	1		
Age in months						
2–4 months	100	48	(48.0)	1.6	(0.8–2.9)	0.122
5–7 months	26	8	(30.8)	1		
8–12 months	14	10	(71.4)	2.3	(1.2–4.5)	0.018
Farm Characteristics						
Annual production turnover						
30–100	50	27	(54.0)	1.8	(0.7–4.8)	0.189
101–300	80	36	(45.0)	1.5	(0.6–4.0)	0.396
>300	10	3	(30.0)	1		
Source of farm feed						
Commercial	10	7	(70.0)	1.6	(1.0–2.5)	0.151
Self-made	100	45	(45.0)	1		
Both	30	14	(46.7)	1.0	(0.7–1.6)	0.87
Change of farm feed from 2022						
Yes	50	30	(60.0)	1.5	(1.1–2.1)	0.025
No	90	36	(40.0)	1		
Antimicrobial usage						
For treatment only	60	26	(43.3)	1.7	(0.8–3.9)	0.155
For prophylaxis only	50	28	(56.0)	2.2	(1.0–5.0)	0.021
For both prophylaxis and treatment	20	5	(25.0)	1		
No antimicrobials for prophylaxis or treatment	10	7	(70.0)	2.8	(1.2–6.6)	0.027
Frequency of prophylaxis *						
Monthly	40	18	(45.0)	1		
Every two months	20	9	(45.0)	1	(0.6–1.8)	0.998
Every three months	10	6	(60.0)	1.5	(0.7–2.5)	0.424
Vet consulted for treating sick pigs *						
Yes	40	15	(37.5)	1		
No	40	16	(40.0)	1.1	(0.6–1.9)	0.823

Footnotes: MDR = multi-drug resistance; PR = prevalence ration; CI = confidence intervals; Vet = veterinary officer. * not all farms used antimicrobials as prophylaxis and therefore the numbers do not add up to 140.

## Data Availability

Requests to access the data from the study should be sent to the corresponding author.

## Supplementary Materials

The following supporting information can be downloaded at: https://www.mdpi.com/article/10.3390/tropicalmed10090266/s1, Figure S1: Polymerase chain reaction (PCR) amplification of mcr-1 gene for colistin resistance in Enterobacterales; Table S1: Dissemination activities following first operational research on healthy pigs in Greater Accra, Ghana, 2022; Table S2: Recommendations from first operational research conducted in healthy pigs in Greater Accra, Ghana, 2022, and status of actions.
