# Supplementary material for: Prevalence of Antimicrobial and Colistin Resistance in Enterobacterales in Healthy Pigs in Ghana Before and After Farmer Education
Source: Trop Med Infect Dis. 2025 Sep 17;10(9):266. doi: 10.3390/tropicalmed10090266 (PMC12474154; doi:10.3390/tropicalmed10090266)
Supplement: Supplementary file 1 [file tropicalmed-10-00266-s001.zip › SUPPLEMENTARY MATERIALS S1.pdf]

SUPPLEMENTARY MATERIALS S1:

Dissemination activities and recommendations following the first operational research study on healthy pigs in greater Accra, Ghana, 2022

Table S1. Dissemination activities following first operational research on healthy pigs in Greater Accra, Ghana, 2022.

Type of Dissemination	Who Received	Where conducted	Dissemination tools used	When conducted
Mid-year scientific review	Animal Research Scientists (50)	Animal Research Institute	10-minute PPT	July 2022
Published Paper	3301 views [A]<br>7 citations	IJERPH	Open Access<br><br>Institutional website (CSIR-Animal research)	September 2022
National AMR Committee Meeting	AMR committee Members	SORT IT Module 4	3-minute PPT	October 2022
One Health Farmers Meeting	Farmers included in study and others in Accra, representatives from FAO, WHO, NADMO and staff of CSIR-Animal Research Institute	CSIR-Animal Research Institute	Plain language hand-out<br><br>10-minute PPT	November 2022
Face-to-face meeting	Farmers, livestock extension officers and Community members	Accra	Plain language hand-out	November 2022
Animal Research Institute annual report	All (13) CSIR Institutes	Ghana	Paper abstract	December 2022
Program Dissemination Seminar	Multiple stakeholders including the VSD	WHO/TDR GHANA SORT IT Program	10-minute PPT	July 2023

Footnotes: PPT = power point presentation; IJERPH = International Journal of Environmental Research and Public Health; AMR = antimicrobial resistance; SORT IT = Structured Operational Research Training Initiative; CSIR = Council for Scientific and Industrial Research; WHO/TDR = UN Special Programme for Research and Training in Tropical Diseases; FAO = Food and Agriculture Organization of the United Nations; NADMO = National Disaster Management Organization; VSD = Veterinary Services Directorate

[A] as of 18th July 2025

Table S2. Recommendations from first operational research conducted in healthy pigs in Greater Accra, Ghana, 2022, and status of actions.

Recommendation	Action Status	Site of Action
Set up resistance surveillance programs in other regions of Ghana	Not implemented	-
Provide education for the farmers and livestock handlers about human and animal hygiene practices, biosafety and biosecurity measures, proper antimicrobial use, and how to ensure good animal husbandry	Implemented	Study farms and other farms in Accra
Conduct research into antimicrobial residues in farm feed, farm water and farm dust	Implemented	Study farms in Accra
Strengthen regulatory policy on antimicrobial usage and monitoring in the animal industry	Not implemented	-
