# Supplementary material for: Prevalence of Antimicrobial and Colistin Resistance in Enterobacterales in Healthy Pigs in Ghana Before and After Farmer Education
Source: Trop Med Infect Dis. 2025 Sep 17;10(9):266. doi: 10.3390/tropicalmed10090266 (PMC12474154; doi:10.3390/tropicalmed10090266)
Supplement: Supplementary file 1 [file tropicalmed-10-00266-s001.zip › SUPPLEMENTARY MATERIALS S2.pdf]

SUPPLEMENTARY MATERIALS S2:

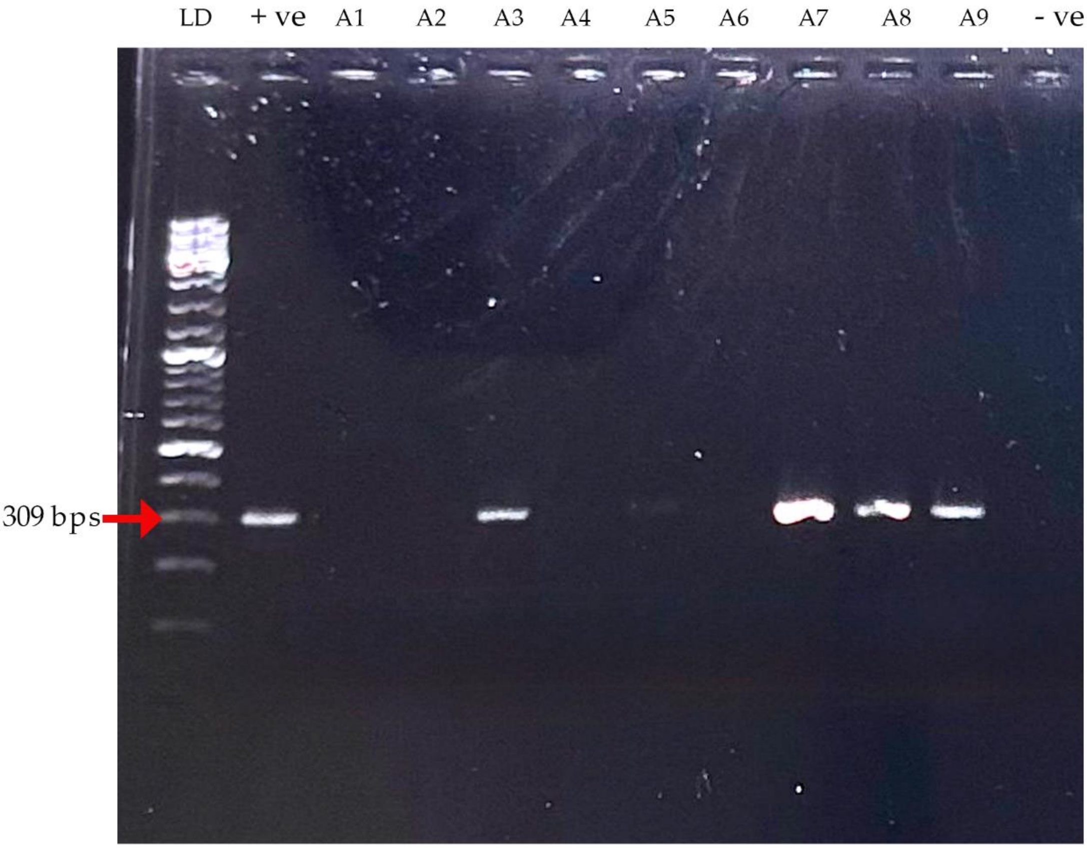

Figure S1. Polymerase chain reaction (PCR) amplification of *mcr*-1 gene for colistin resistance in Enterobacterales.
